# Comparative Analysis of Pain Behaviours in Humanized Mouse Models of Sickle Cell Anemia

**DOI:** 10.1371/journal.pone.0160608

**Published:** 2016-08-05

**Authors:** Jianxun Lei, Barbara Benson, Huy Tran, Solomon F. Ofori-Acquah, Kalpna Gupta

**Affiliations:** 1 Vascular Biology Center, Division of Hematology, Oncology and Transplantation, Department of Medicine, University of Minnesota, Minneapolis, Minnesota, United States of America; 2 Division of Hematology/Oncology, Department of Medicine, University of Pittsburgh, Pittsburgh, Pennsylvania, United States of America; 3 Center for Translational and International Hematology, Vascular Medicine Institute, University of Pittsburgh, Pittsburgh, Pennsylvania, United States of America; Boston Children’s Hospital and Harvard Medical School, UNITED STATES

## Abstract

Pain is a hallmark feature of sickle cell anemia (SCA) but management of chronic as well as acute pain remains a major challenge. Mouse models of SCA are essential to examine the mechanisms of pain and develop novel therapeutics. To facilitate this effort, we compared humanized homozygous BERK and Townes sickle mice for the effect of gender and age on pain behaviors. Similar to previously characterized BERK sickle mice, Townes sickle mice show more mechanical, thermal, and deep tissue hyperalgesia with increasing age. Female Townes sickle mice demonstrate more hyperalgesia compared to males similar to that reported for BERK mice and patients with SCA. Mechanical, thermal and deep tissue hyperalgesia increased further after hypoxia/reoxygenation (H/R) treatment in Townes sickle mice. Together, these data show BERK sickle mice exhibit a significantly greater degree of hyperalgesia for all behavioral measures as compared to gender- and age-matched Townes sickle mice. However, the genetically distinct “knock-in” strategy of human α and β transgene insertion in Townes mice as compared to BERK mice, may provide relative advantage for further genetic manipulations to examine specific mechanisms of pain.

## Introduction

Sickle cell anemia (SCA) is characterized by unpredictable and recurrent episodes of acute pain during vaso-occlusive crises, which can be superimposed on chronic pain [1, 2]. Opioids remain the mainstay of therapy, despite the liabilities such as constipation, pruritis, respiratory depression and fear of opioid addiction [3]. Therefore, effective analgesics devoid of side effects are required for the management of pain in SCA. The understanding of mechanisms of sickle pain and development of more effective analgesics to treat sickle pain remain an unmet need.

Transgenic sickle mice offer the opportunity to examine mechanisms of pain in SCA. We have shown that transgenic homozygous BERK mice expressing >99% human sickle hemoglobin (HbS), exhibit both pain and neurochemical changes similar to humans with SCA [4–20]. In contrast, transgenic mice expressing mouse globin chains along with human HbS, including NY1DD and S+S^Ant^, show modest or no chronic, tonic hyperalgesia [4]. Incitement of hypoxia/reoxygenation (H/R) in S+S^Ant^ did not significantly increase mechanical or thermal hyperalgesia as compared to control C57BL/6 mice [4]. Mechanical threshold and heat sensitivity were significantly different between HbAA-BERK control mice that express exclusively normal human hemoglobin (HbA) and C57BL/6 mice, the genetic background strain of NY1DD and S+S^Ant^ mice. Thus, pain behaviors in mice can be influenced by the presence of different globin chains and genetic background.

Commonly used mouse models that express human HbS without mouse α and β chains are BERK and Townes transgenic sickle mice [21–23]. Of the two Townes sickle transgenic models, we used the “knock-in” model where human α and β chains are inserted (knocked-in) in place of mouse α and β chains [23]. These 2 models exhibit severe features of SCA including hematologic disease, organ damage and foreshortened life-span similar to the clinical disease [4, 21–25]. Both Townes and BERK homozygous mice exhibit inflammation, oxidative stress, and endothelial activation, similar to the pathobiology reported for patients with SCA [26–29]. BERK mice have knock-out genes for the endogenous mouse hemoglobin genes and a transgene for the expression of human hemoglobin chains. Townes mice carry human globin knock-in genes replacing the endogenous mouse genes. Therefore, both these mice appear to be suitable models to analyze pain mechanisms in SCA. BERK sickle mice have been most commonly used to examine the characteristics and mechanisms of pain in SCA [4–9, 26, 30–33]. Use of Townes sickle mice is emerging to examine hyperalgesia [34, 35]. A previous study comparing BERK and Townes mice showed sensitization of peripheral sensory nerve fibers varied according to strain, sex, age, and mouse genotype; and was distinct from that observed in pain models of neuropathic and inflammatory pain [35]. In another study, dexmedetomidine decreased heat sensitivity and deep tissue hyperalgesia in both mouse strains but differentially affected sensitization of peripheral sensory nerve fibers, Aδ-fibers in HbSS-BERK and C-fibers in Townes [34].

It is therefore reasonable to understand the comparative differences between the two mouse models (BERK and Townes) with respect to different characteristics of pain, age and gender. To facilitate future investigation on pain in SCA, the present study compared the mechanical, thermal, and deep tissue hyperalgesia in HbSS-BERK and HbSS-Townes sickle mice, as well as HbAA-BERK and HbAA-Townes control mice expressing normal human hemoglobin A on their respective genetic background. In addition, pain due to vaso-occlusive crises (VOC) remains a major cause of hospitalization and challenge to treat; our study therefore also examined the potential of using Townes mice for evoking hyperalgesia after hypoxia-reoxygenation (H/R) treatment.

## Methods

### Animals

#### Sickle (HbSS-BERK; HbSS-Townes) and control mice (HbAA-BERK; HbAA-Townes)

Townes mice were bred in Dr Ofori-Acquah’s laboratory at the University of Pittsburgh, using breeding pairs from Jackson Laboratories (Stock No: 013071, Townes model, hα/hα::β^A^/β^S^, hα/hα::-383 γ-β^A^/-1400 γ-β^S^). Some Townes mice were bred in Gupta laboratory at the University of Minnesota using breeders from the Ofori-Acquah laboratory. The Townes mouse “knock-in” model of SCA used in this study was developed by replacing the mouse α globin genes with a human-globin gene (hα/hα) and by replacing the mouse beta globin genes with human A-γ- and β-S-globin genes (-1400 γ-βs/-1400 γ-βs) [23]. HbSS-Townes mice have severe hemolytic anemia due to erythrocyte sickling, reticulocytosis, splenic infarcts, kidney damage, and overall poor health [23]. HbSS-BERK do not express any mouse hemoglobin and carry copies of a transgene containing human α1, γ, δ, and β-sickle genes on a highly mixed genetic background [21]. HbSS-BERK have severe disease that simulates human sickle cell anemia including hemolysis, reticulocytosis, anemia, extensive organ damage, shortened life span and pain [4, 5, 21]. Control HbAA-BERK are littermates of HbSS-BERK and therefore have the same mixed genetic background as HbSS-BERK, but exclusively express normal human hemoglobin A (human α and β globins) and no murine globins. Similarly, Townes control mice do not express mouse hemoglobin and express the human globin gene (hα/hα), human A-γ- and human wild-type β-globin (hβ/hβ) genes [23, 25].

Mice were bred and phenotyped for sickle and normal human hemoglobin by isoelectric focusing as previously described [5]. Genotyping for the knockout and hemoglobin transgenes was done by Transnetyx (Cordova, TN). Nine groups of female mice of varying ages were studied: HbSS-Townes, 4.12 ± 0.12 months, 7.19 ± 0.12 months and 10.18 ± 0.41 months; HbAA-Townes, 3.69 ± 0.0.20 months and 8.94 ± 0.19; and HbSS-BERK, 4.42 ± 0.05 months, 6.80 ± 0.09 months and 10.80 ± 0.63 months; HbAA-BERK, 8.97 ± 0.0.18 months. Seven groups of male mice of varying ages were studied: HbSS-Townes, 5.98 ± 0.66 months and 9.99 ± 0.07 months, HbAA-Townes, 5.93 ± 0.55 months and 9.34 ± 0.37 months; HbSS-BERK, 6.39 ± 0.32 months and 10.80 ± 0.63 months; and HbAA-BERK: 11.00 ± 0.99 months.

### Ethics Statement

All experiments were performed following approved protocols from the University of Minnesota’s Institutional Animal Care and Use Committee and conform to the statutes of the Animal Welfare Act and the guidelines of the Public Health Service as issued in the Guide for the Care and Use of Laboratory Animals.

### Pain-related behaviors

Mice were acclimatized to each test protocol in a quiet room at constant temperature and tested for thermal- (heat and cold), mechanical-, and deep tissue-hyperalgesia (grip force) as described [5].

#### Mechanical hyperalgesia

Mechanical hyperplasia was measured by the paw withdrawal frequency (PWF) evoked by 10 consecutive applications of a 1.0 g (4.08 mN) von Frey (Semmes-Weinstein) monofilament (Stoelting Co., Wood Dale, IL) to the plantar surface of each hind paw for 1–2 seconds with a force sufficient to bend the filament. An inter-stimulus interval of at least 5 seconds was observed. Only vigorous withdrawal responses were counted; increased PWF indicates increased hyperalgesia.

#### Thermal hyperalgesia

For heat sensitivity, a radiant heat stimulus was applied to the plantar surface of the hind paw with a projector lamp bulb (CXL/CXR, 8 V, 50 W). Paw withdrawal latency (PWL) to the nearest 0.1 second was recorded when the mouse withdrew its paw from the stimulus; shorter interval indicates increased heat sensitivity. For cold sensitivity, the number of times mice lifted or rubbed the forepaws together (PWF) on a cold plate (4°C) over a period of 2 minutes was determined; a lower value indicates increased cold sensitivity.

#### Grip force

To assess deep tissue hyperalgesia, peak forepaw grip force was measured using a computerized grip force meter (SA Maier Co., Milwaukee, WI). Mice held by the tail were made to pull on a wire-mesh gauge with their forepaws and gradually pulled by the tail. The force (in g) exerted at the gauge at the time of grip release by the mouse was recorded as the grip force. Deep tissue hyperalgesia was defined as a decrease in the grip force.

### Hypoxia/Reoxygenation

Mice were exposed to hypoxia with 8% O_2_ and 92% N_2_ for 3h followed by re-oxygenation at room air for 1h as described [4].

### Statistical analysis

**Fig 1:**
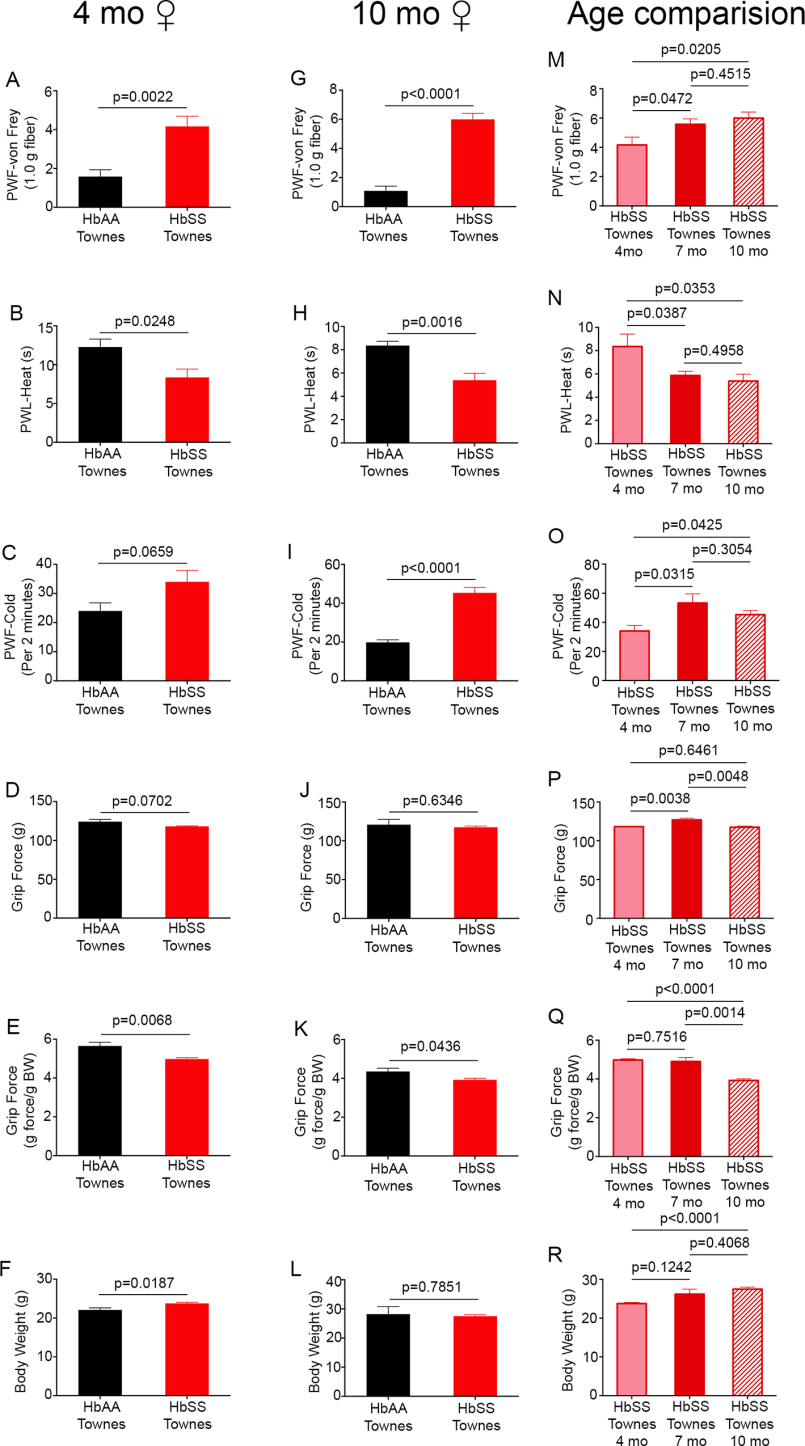
Female Townes sickle mice exhibit chronic hyperalgesia. Chronic hyperalgesia is defined as an enhanced sensitivity to mild nociceptive stimuli in the absence of any source that evokes injury. (A-L) Female HbSS-Townes mice compared to age-matched female HbAA-Townes mice at 4 months (A-F) and 10 months (G-L). Age in months ± SEM for HbSS-Townes was 4.12 ± 0.12 (n = 6) and 10.18 ± 0.41 (n = 6); for HbAA-Townes was 3.67 ± 0.19 (n = 6) and 8.94 ± 0.19 (n = 6). (M-R) Effect of age on hyperalgesia in Townes female sickle mice. Age in months ± SEM for female HbSS-Townes mice was 4.12 ± 0.12 (n = 6) at 4 months, 7.19 ± 0.12 (n = 7) at 7 months, and 10.18 ± 0.41 (n = 6) at 10 months. Mechanical hyperalgesia (A, G, M), thermal sensitivity to heat (B, H, N) and cold (C, I, O), deep tissue hyperalgesia (D-E, J-K, P-Q), and body weight (F, L, R) are shown. Data are presented as mean ± SEM. PWF, paw withdrawal frequency; PWL, paw withdrawal latency; BW, body weight.

**Fig 2:**
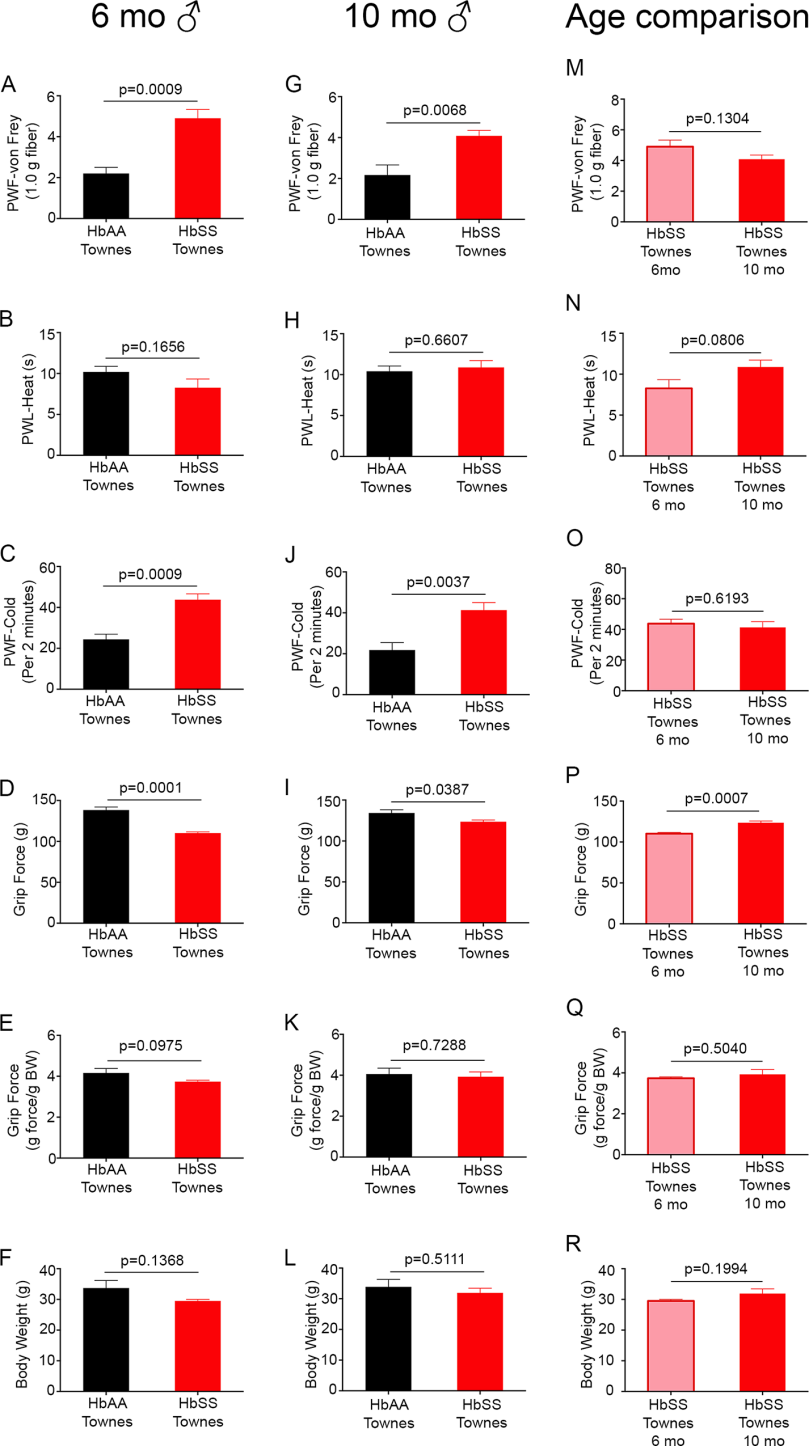
Male Townes sickle mice exhibit chronic hyperalgesia. Male HbSS-Townes mice compared to age-matched male HbAA-Townes mice at 6 months (A-F) and 10 months (G-L). (M-R) Effect of age on hyperalgesia in Townes male sickle mice. Age in months ± SEM for male HbSS-Townes mice was 5.99 ± 0.66 (n = 6) at 6 months and 9.99 ± 0.07 (n = 6) at 10 months. Age in months ± SEM for male HbAA-Townes mice was 5.93 ± 0.55 (n = 6) at 6 months and 9.34 ± 0.37 (n = 6) at 10 months. (A, G, M) Mechanical hyperalgesia, thermal sensitivity to (B, H, N) heat and (C, I, O) cold, (D-E, J-K, P-Q) deep tissue hyperalgesia and (F, L, R) body weight are shown. Data are presented as mean ± SEM. PWF, paw withdrawal frequency; PWL, paw withdrawal latency; BW, body weight.

**Fig 3:**
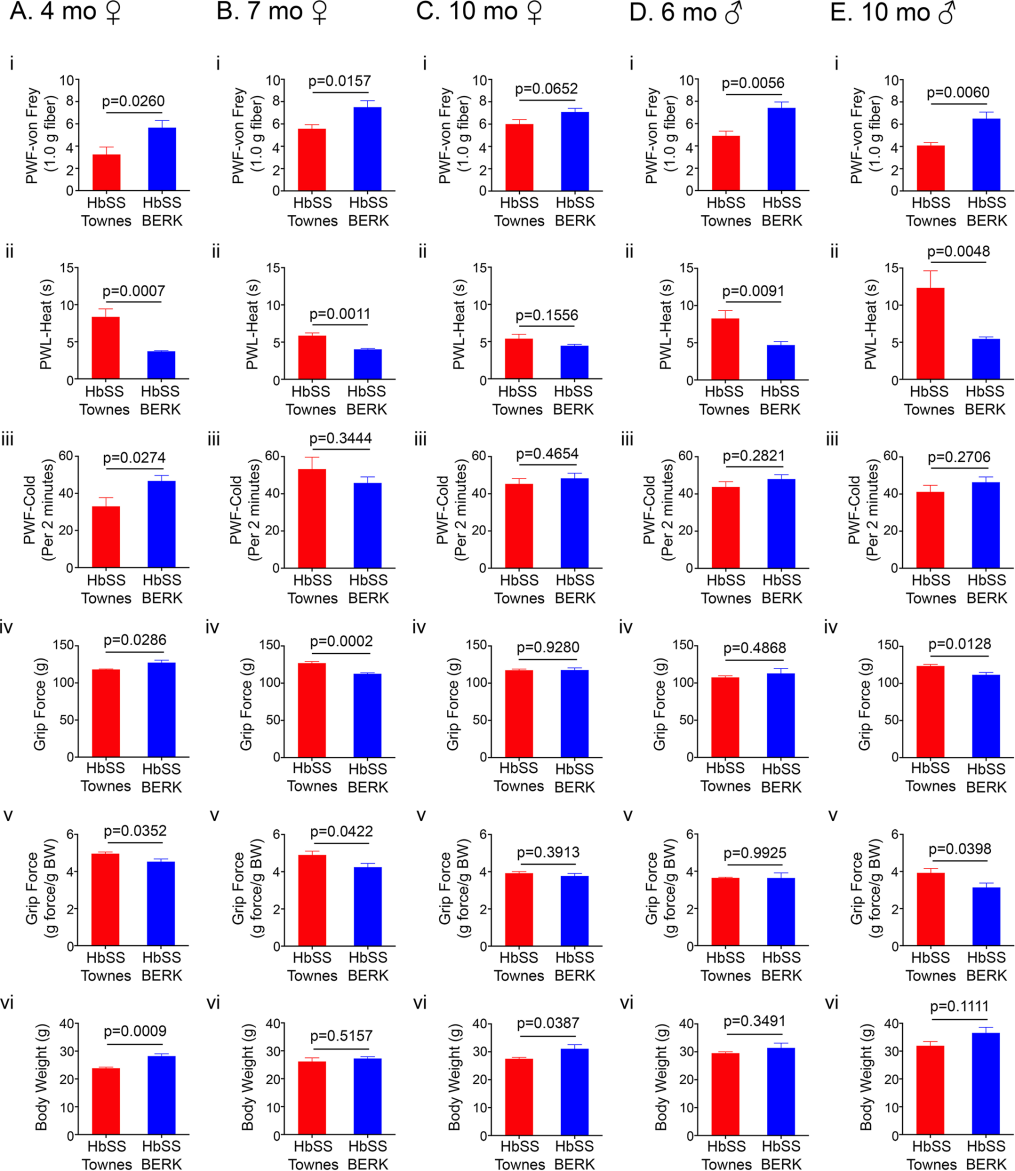
Chronic hyperalgesia in Townes sickle mice compared to BERK sickle mice. Hyperalgesia in female HbSS-Townes mice compared to age-matched female HbSS-BERK mice at 4 months (A), 7 months (B), and 12 months (C). Age in months ± SEM for female HbSS-Townes was 4.12 ± 0.12 (n = 6), 7.19 ± 0.12 (n = 7), and 10.18 ± 0.41 (n = 6), and for female HbSS-BERK was 4.42 ± 0.05 (n = 7), 6.80 ± 0.09 (n = 6), and 10.80 ± 0.63 (n = 6). Comparison of hyperalgesia between male Townes sickle mice and age-matched male BERK sickle mice at 6 months (D) and 10 months (E). Age in months ± SEM for male HbSS-Townes was 5.99 ± 0.66 (n = 6) and 9.99 ± 0.07 (n = 6) and for male HbSS-BERK was 6.39 ± 0.32 (n = 6) and 10.96 ± 0.14 (n = 8). (i) Mechanical hyperalgesia, thermal sensitivity to (ii) heat and (iii) cold, (iv-v) deep tissue hyperalgesia, and (vi) body weight are shown. Data are presented as mean ± SEM. PWF, paw withdrawal frequency; PWL, paw withdrawal latency; BW, body weight.

**Fig 4:**
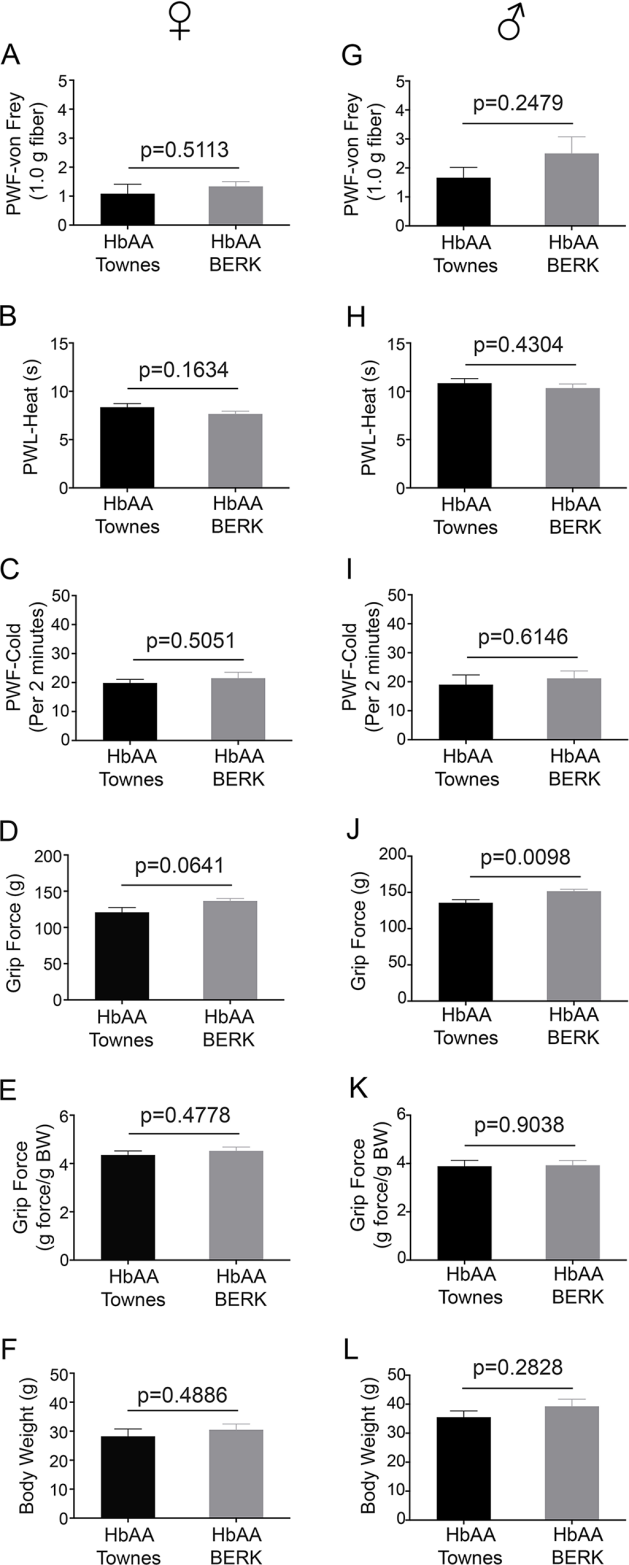
Pain behaviors in control Townes and BERK mice. Mechanical hyperalgesia (A, G), thermal sensitivity to heat (B, H) and cold (C, I), deep tissue hyperalgesia (D-E, J-K) and body weight (F, L) for female HbAA mice (A-F) and male HbAA mice (G-L) are shown. Mean age in months ± SEM for each group was as follows: female HbAA-Townes: 8.94 ± 0.19 (n = 6), female HbAA-BERK: 8.97 ± 0.18 (n = 6), male HbAA-Townes: 9.34 ± 0.37 (n = 6), and male HbAA-BERK: 11.00 ± 1.0 (n = 6). PWF, paw withdrawal frequency; PWL, paw withdrawal latency; BW, body weight.

**Fig 5:**
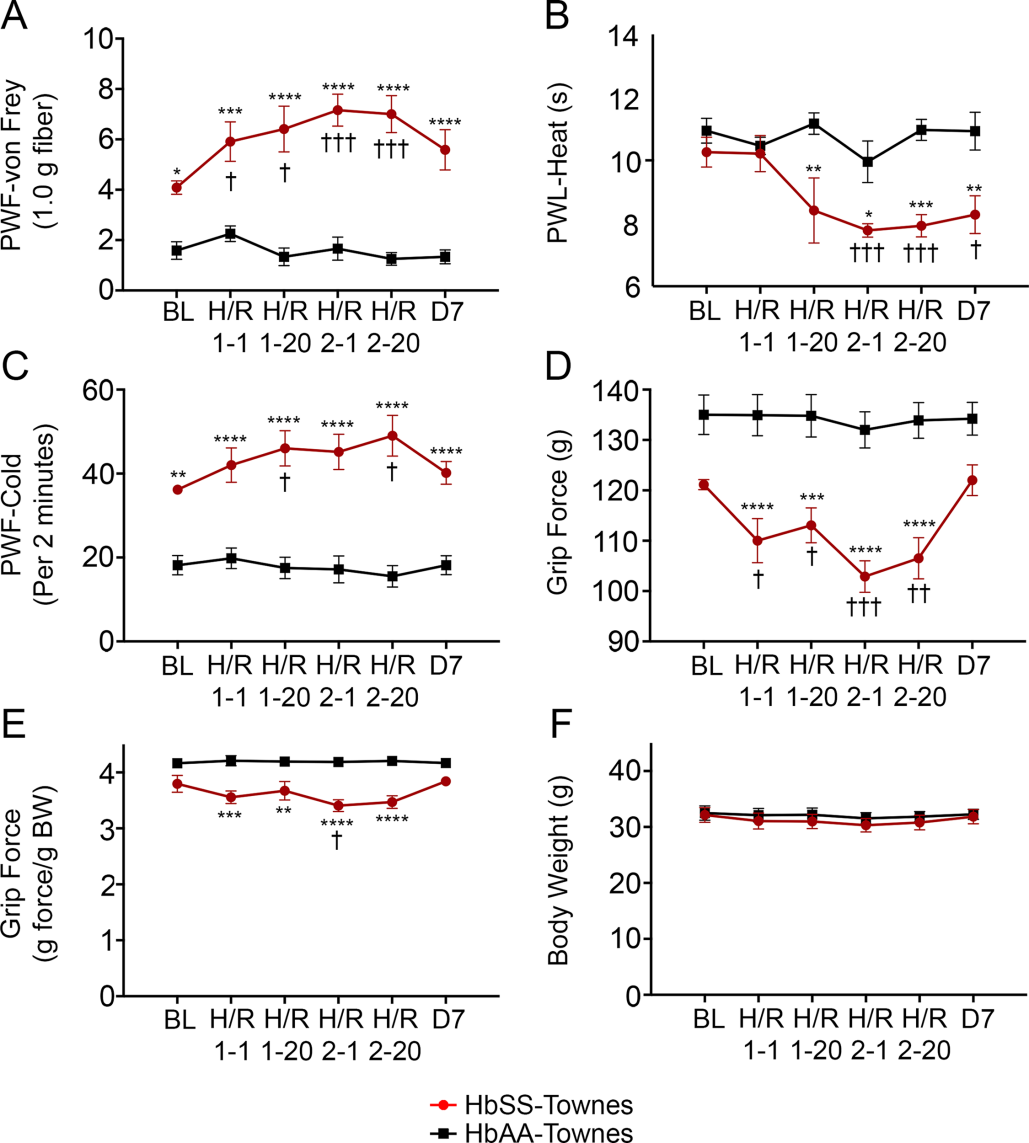
Hypoxia/reoxygenation induces acute hyperalgesia in Townes sickle mice. After baseline pain determination, male HbSS-Townes mice and HbAA-Townes mice were exposed to hypoxia (H) for 3h, followed by reoxygenation (R) in room air for 1h. The H/R treatment was then repeated 24h later. Mechanical hyperalgesia (A), thermal sensitivity to heat (B) and cold (C), and deep tissue hyperalgesia (D-E) and body weight (F) were assessed at 1h (H/R 1–1) and 20h (H/R 1–20) after the first H/R; at 1h (H/R 2–1), 20h (H/R 2–20) and 7 days (D7) after the second H/R. Mean age in months ± SEM for each group of mice was as follows: HbSS-Townes: 9.99 ± 0.07 (n = 6) and HbAA-Townes: 9.34 ± 0.37 (n = 6). *p < 0.05, **p < 0.01 ***p < 0.001 for HbSS-Townes vs. HbAA-Townes at respective time point; ^†^*p* < 0.05, ^††^*p* < 0.01, ^†††^*p* < 0.001 vs. baseline (BL) of corresponding group. Each data point is presented as mean ± SEM. PWF, paw withdrawal frequency; PWL, paw withdrawal latency; BW, body weight.

All data were analyzed using Prism software (v 6.0e, GraphPad Prism Inc., San Diego, CA). Unpaired t-test was used to compare pain behaviour between groups (Figs 1–4). For responses after H/R treatments (Fig 5) for between-group comparisons over time, all data were compared by two-way ANOVA analysis of variance with Bonferroni’s multiple comparison. For within-group comparisons over time, all data were compared using 1-way ANOVA analysis of variance with Bonferroni’s multiple comparison. A *p*-value of < 0.05 was considered significant. All data values are presented as mean ± SEM.

## Results

### Chronic hyperalgesia in female Townes sickle mice

We observed that ~4-month-old female HbSS-Townes mice showed increased mechanical, heat (p< 0.05; Fig 1A and 1B), and deep tissue hyperalgesia (p< 0.01; Fig 1E), but no significant difference in cold sensitivity (p> 0.05; Fig 1C), as compared to age- and gender-matched HbAA-Townes control mice. Similarly, when compared to age- and gender-matched controls, 10-month-old female HbSS-Townes mice showed increased mechanical (p< 0.0001; Fig 1G), heat (p< 0.01; Fig 1H), and deep tissue hyperalgesia (p< 0.05; Fig 1K). In addition, the 10-month-old female HbSS-Townes mice exhibited a significant difference in cold sensitivity compared to controls (p< 0.0001; Fig 1I). Grip force when expressed per mouse did not show a significant difference between sickle and control Townes mice for either 4- or 10-month-old mice (p> 0.07 and p> 0.6; Fig 1D and 1J, respectively). However, upon correcting for weight, by expressing per gram of body weight, a significantly lower grip force was observed in HbSS-Townes as compared to control (p< 0.01 and p< 0.05; Fig 1E and 1K, respectively). Therefore, female Townes sickle mice show most of the characteristics of hyperalgesia observed in SCA except cold hyperalgesia in the youngest group.

### Effect of age on hyperalgesia in female Townes sickle mice

We measured pain behaviors longitudinally in the same Townes female mice from 4–7 and 10 months of age. With increasing age, mechanical, heat, and cold hyperalgesia (p< 0.05; Fig 1M–1O) increased at ~7 and ~10 months of age in female HbSS-Townes as compared to their measurements at ~4 months of age. Surprisingly, female Townes sickle mice at 10 months of age did not have a further increase in mechanical and thermal hyperalgesia in comparison to 7-month-old Townes sickle mice (p> 0.3; Fig 1M–1O). Therefore, HbSS-Townes mice show the same characteristic features of pain in SCA as do HbSS-BERK mice, with the exception of a delay in the development of cold hyperalgesia (Fig 1C). Surprisingly, significantly increased grip force was observed when expressed per mouse for ~7-month-old HbSS-Townes as compared to ~4-month-old HbSS-Townes (p< 0.005; Fig 1P), but a significantly lower grip force value was observed when comparing 7-month-old to 10-month-old HbSS-Townes (p< 0.005; Fig 1P), and there was no difference between 4-month-old and10-month-old HbSS-Townes (p> 0.6; Fig 1P). In contrast, grip force did not change significantly between 4 to 7 months when grip force was expressed per gram body weight (p> 0.75; Fig 1Q and 1R), perhaps due to an increase in their body weight with age. However, a significantly lower grip force per gram of body weight was observed in 10-month-old female HbSS-Townes as compared to either 4- or 7-month-old female HbSS-Townes (p< 0.0001 and p< 0.005, respectively; Fig 1Q).

### Increased hyperalgesia in male Townes sickle mice as compared to control mice

In contrast to female HbSS-Townes mice, we observed that both 6- and 10-month-old male HbSS-Townes mice showed increased mechanical (p< 0.001 and p< 0.01; Fig 2A and 2G, respectively) and cold hyeralgesia (p< 0.001 and p< 0.005; Fig 2C and 2J, respectively), but no significant difference in heat sensitivity (p> 0.1 and p> 0.6; Fig 2B and 2H, respectively), as compared to age- and gender-matched HbAA-Townes control mice. Grip force when expressed per mouse showed a significant difference between sickle and control Townes mice at either 6 or 10 months of age (p = 0.0001 and p<0.05; Fig 2D and 2I, respectively). However, upon correcting for weight, by expressing per gram of body weight, no significant difference in grip force was observed in male HbSS-Townes as compared to control (p> 0.09 and p> 0.7; Fig 2E and 2K, respectively). Therefore, male Townes sickle mice show only the characteristics of mechanical and cold hyperalgesia observed in SCA.

When pain behaviors in male Townes sickle mice at 6 and 10 months of age were compared, we found no significant difference in mechanical, thermal, and deep tissue hyperalgesia with increasing age (p> 0.08; Fig 2M–2O and 2Q), In contrast, when grip force was expressed per mouse, the value was significantly increased at 10 months in comparison to 6-month-old male Townes sickle mice (p< 0.001; Fig 2P). Female HbSS-Townes mice at 7 months (Fig 1M–1O) in comparison to the male HbSS Townes at 6 months (Fig 2M–2O) show increased mechanical (PWF of 5.57 ± 0.37 vs 4.90 ± 0.43, respectively), heat hyperalgesia (PWL of 5.86 ± 0.37 vs 8.27 ± 1.05, respectively), and cold hyperalgesia (PWF of 53.3 ± 6.4 vs 43.8 ± 2.8, respectively). However, deep tissue hyperalgesia, expressed per gram body weight, is increased in Townes male sickle mice at 6 months compared to females at 7 months (g force/ g B.W. of 4.90 ± 0.21 vs 3.74 ± 0.07; Fig 1Q and Fig 2Q). Similarly, female HbSS-Townes mice at 10 months (Fig 1M and 1N) in comparison to male HbSS Townes at 10 months (Fig 2M and 2N) had increased mechanical (PWF of 6.00 ± 0.41 vs 4.08 ± 0.27, respectively) and heat hyperalgesia (PWL of 5.39 ± 0.37 vs 10.88 ± 0.84, respectively), while cold hyperalgesia (PWF of 45.3 ± 2.8 vs 41.8 ± 3.7; Figs 1O and 2O, respectively) and deep tissue hyperalgesia were similar at 10 months (g force/ g B.W. of 3.92 ± 0.09 vs 3.93 ± 0.24; Figs 1Q and 2Q, respectively). In summary, female Townes sickle mice exhibit more mechanical and heat hyperalgesia than age-matched male Townes sickle mice.

### Comparative differences in hyperalgesia between BERK and Townes sickle mice

Gender (female) and age-matched BERK sickle mice, exhibited significantly greater degree of hyperalgesia for all behavioral measures tested when compared to Townes sickle mice at ~4 months of age (mechanical, cold and deep tissue, p< 0.05; heat, p< 0.001; Fig 3A-i to 3A-v). Interestingly, BERK female sickle mice at ~4 and 10 months of age weighed significantly more than age/gender-matched Townes mice (p = 0.0009 and 0.0387, respectively; Fig 3A-vi and 3C vi). Consistent with their increased weight, BERK mice showed increased grip force as compared to Townes mice (p< 0.05; Fig 3A-iv). However, when grip force was expressed as per gram body weight to correct for increased body weight, BERK mice showed a significantly decreased grip force as compared to Townes mice (p< 0.05; Fig 3A-v). Female BERK sickle mice at 7 months still exhibited significantly more hyperalgesia for all nociceptive measures tested than female Townes mice (p< 0.5 Fig 3B) except cold hyperalgesia (p> 0.3; Fig 3B-iii). In contrast, male BERK sickle mice at 6 and 10 months exhibited significantly more mechanical and heat hyperalgesia (p< 0.01; Fig 3D-i and 3D-ii and 3E-i and 3E-ii); cold hyperalgesia was similar at 6 months and appeared modestly increased at 10 months when compared to male Townes (p> 0.25; Fig 3D-iii and 3E-iii). Deep tissue hyperalgesia in male BERK sickle mice was similar at 6 months and significantly increased at 10 months when compared to male Townes mice (p = 1 and p< 0.05, Fig 3D-v and 3E-v, respectively). Overall, BERK mice show significantly increased hyperalgesia as compared to age- and gender-matched Townes mice. Importantly, chronic hyperalgesia starts earlier in age in BERK sickle mice.

### Townes- and BERK-specific control mice do not show changes in behaviour testing

We found no difference in the pain behaviour responses among either female or male HbAA-Townes and HbAA-BERK control mice at 10 months (mechanical, thermal, and deep tissue hyperalgesia, p> 0.1620; Fig 4A–4C, 4E and Fig 4G–4I) unless expressed as grip force per mouse (p< 0.07 and p< 0.01; Fig 4D and 4J, respectively). Correcting for body weight is important considering that increase in weight may lead to increased muscular strength, which may in turn influence the grip force as seen in Townes mice (Figs 1P and 4R, Figs 2P and 4R, Fig 4J and 4L).

### Hypoxia/reoxygenation evokes acute hyperalgesia in Townes sickle mice

We found that mechanical hyperalgesia and cold sensitivity increased after 1 h of reoxygenation (H/R1-1) and remained increased at 20 h (H/R1-20) in Townes sickle mice as compared to their baseline (p< 0.05) and to Townes control mice (p< 0.01; Fig 5A and 5C). The second exposure of Townes sickle mice to hypoxia (H/R2) caused a slight increase of mechanical hyperalgesia after 1 h of reoxygenation (H/R2-1, p< 0.001; Fig 5A) but cold sensitivity increased at 20 h (H/R2-20, p< 0.05; Fig 5C). Heat sensitivity did not increase until 20 h (H/R1-20) after the first H/R treatment, while H/R2 caused further increase of sensitivity after 1 h of reoxygenation (H/R2-1, p< 0.001) which remained elevated at 20 h (H/R2-20, p< 0.001) and after 7 days (p< 0.05; Fig 5B). Grip force adjusted for body weight for Townes sickle mice was only significantly affected after the second episode of H/R compared to baseline (Fig 5E, p< 0.05). In comparison to the Townes control mice, H/R treatment of Townes sickle mice caused a decrease in grip force adjusted for body weight which remained lower for the next 20 h (p< 0.01, Fig 5E).

## Discussion

The present study compared mechanical, deep tissue and thermal hyperalgesia in the homozygous HBSS-Townes and HbSS-BERK mouse models expressing >99% human sickle hemoglobin. Importantly, we used age-matched control mice for each model with genetic background and transgene induction manipulations similar to the respective sickle mice. Comparisons were also made between females and male HbSS-Townes and HbSS-BERK, because of gender-dependent variability in hyperalgesia, which has been shown to increase with age in HbSS-BERK mice [5]. Therefore, in the present study, we also compared the effect of age on progression of hyperalgesia in HbSS-Townes mice. In addition to tonic/chronic hyperalgesia, we also characterized the potential of evoking H/R-incited hyperalgesia in HbSS-Townes mice.

Most pain studies have focused on male rodents due to the variability associated with the menstrual cycle. It is becoming widely recognized that mechanisms of pain may vary between male and females [36–38]. Spinal TLR4 mediated inflammatory hyperalgesia has been suggested to be involved in males but not in female mice [38]. We found that spinal TLR4 transcripts are increased in BERK sickle male mice, and TLR4 has been demonstrated to play a critical role in sickle pathobiology [5, 27, 39]. We show that Townes female sickle mice have increased hyperalgesia as compared to male sickle mice (Figs 1 and 2) as we previously observed for BERK sickle mice [5]; this difference is consistent with the painful episodes in patients, which are significantly longer, higher in intensity, and spread over a larger body surface area in female as compared to male patients [10, 11]. We therefore believe that it is critical to examine pain mechanisms in females. Pain in females and in older mice is an important consideration because female sickle patients and older patients require longer hospital stays and treatment with drugs for neuropathic pain [40]. This could be due to neural injury as shown by us in BERK sickle mice at a relatively early age [5]. It is likely that Townes mice do not have the neural damage to the same extent as in BERK because of expression of γ-globin for a longer duration post-natally as compared to BERK.

We previously observed mechanical, thermal and deep tissue hyperalgesia in BERK sickle mice, which was subsequently shown to occur in patients with SCA upon quantitative sensory testing [4, 5, 12, 41]. Townes sickle mice also show mechanical, thermal and deep tissue hyperalgesia, which are characteristics of hyperalgesia observed in SCA. Although young Townes sickle mice do not exhibit cold sensitivity (4-months in Fig 1C), significantly elevated cold sensitivity is observed in relatively older Townes sickle mice (Fig 1I and 1O). As increased cold sensitivity is a characteristic feature of SCA, relatively older HbSS-Townes mice may be more appropriate to examine sickle pain and associated pathobiology. In another study, Townes mice did not show a significant difference in sensitivity to heat at either younger or older age, but BERK sickle mice demonstrated significantly increased heat sensitivity as compared to C57BL/6 control mice [35]. Townes mice show deep tissue/musculoskeletal hyperalgesia starting earlier in age than other measures (Fig 1E). Previously we demonstrated that deep tissue hyperalgesia was more specific to a sickle state while comparing the response incited by H/R treatment in BERK sickle as compared to normal human hemoglobin expressing control hBERK mice [4]. Moreover, deep tissue hyperalgesia is not an evoked response to a noxious stimuli, rather it reflects the activation of visceral, joint, and musculoskeletal nociceptors, and shows the inherent pain of a mouse and therefore, may be a more insightful determinant of existent sickle-specific hyperalgesia.

In this study, only older male Townes sickle mice show significant increase in mechanical and cold hyperalgesia, while older BERK sickle mice exhibit more hyperalgesia for all nociceptive measures tested (Fig 2G–2L and Fig 3Ei–3Evi). Kenyon et al found that BERK sickle mice have increased heat sensitivity (PWF-heat) that varies according to age and sex and increased cold sensitivity compared to heterozygous BERK, but significantly lower thermal sensitivity compared with C57BL/6 controls [35]. Although C57BL/6 is one of the background strains comprising the BERK lineage, the fact that C57BL/6 has an enhanced cold sensitivity highlights the inappropriateness of using this line as controls in place of the matched HbAA-BERK mouse. Thus, BERK mice of both genders and at different ages demonstrate significantly higher evoked and spontaneous hyperalgesia as compared to Townes mice. We have also observed evidence of spinal nociceptor sensitization in BERK sickle mice, which could be contributing to increased hyperalgesia [42]. Central sensitization is also suggested in humans with SCA [43]. Supportive of spinal nociceptor sensitization, we previously observed activated microglial and astroglial cells and the accompanying increase in reactive oxygen species and substance P in the spinal cords of BERK sickle mice [7]. We observed that central sensitization and persistent pain in HbSS-BERK is accompanied by activation of p42/p44 mitogen activated protein kinase (MAPK)/ extracellular signaling-regulated kinase (ERK) and p38 MAPK signaling [42]. Complementary to central sensitization, Hillery et al observed increased transient receptor potential vanilloid 1 activity in the peripheral nerve terminals in BERK sickle mice compared to control mice [31]. It is therefore likely that attendant peripheral and central nociceptor sensitization contributes to increased tonic hyperalgesia in BERK sickle mice. Mast cell activation with resulting inflammation and neurogenic inflammation mediate sickle pain, which is ameliorated by cannabinoids via both cannabinoid receptors 1 and 2 in HbSS-BERK sickle mice [44]. It remains to be seen whether similar features of peripheral and central sensitization, activation of MAPK, and mast cell activation occur in Townes sickle mice and their response to analgesics. Since behaviors are extremely dependent upon the environment including breeding strategies, diet, temperature, equipment used, handler, etc, subtle differences can be observed in sensitivities between different studies.

It is also likely that some of the hematologic features may account for the relatively less hyperalgesia in Townes mice as compared to BERK. In Townes sickle mice hematocrit was shown to be between 13 and 27% and reticulocytes between 36 and 77% in different lines, as compared to BERK sickle mice with a hematocrit of 28.7 ± 2.5% and 26.8 ± 2.2% reticulocytes [21, 22]. Additionally, BERK sickle mice have excess of α-globin chains as compared to β-S (1.26 ± 0.02) suggestive of slightly β-thalassemic state, but Townes mice show more balanced globin chains. Thus BERK-SS mice have comparatively more severe hematologic phenotype as compared to Townes, which may contribute to increased pain.

Fetal hemoglobin (HbF) is expressed in high concentrations (about 30%–50%) at birth, switching completely to HbS by one month of age in Townes “knockout” SS mice [22]. Although the pattern of hemoglobin switching in Townes knock-in transgenic sickle mice is reported to be similar to Townes knockout SS mice [23], no data are available for HbF levels in the “knock-in” Townes mice. As compared to Townes “knockout” mice, relatively lower (4–26%) HbF is expressed in BERK sickle mice at birth [21]. In sickle patients higher HbF is associated with decreased pain and greater anti-nociceptive intrinsic connectivity in the brain observed by functional magnetic resonance imaging [43]. Therefore, reduced pain at relatively younger age as compared to BERK mice may argue for presumably higher HbF in “knock-in” Townes sickle mice at birth.

BERK sickle mice have been examined for their similarities and differences with human SCA for pathologic manifestations [24]. BERK sickle mice between 1–6 months of age have splenomegaly (not infarcts) with marked splenic hematopoiesis, absence of marrow infarcts and the absence of cerebral infarcts. However, BERK mice have a markedly severe phenotype relative to humans (esp. with a low MCHC and low HbF, as discussed above). This severe pathologic phenotype may underlie increased hyperalgesia in BERK mice.

Painful vaso-occlusive episodes are considered the hallmark of SCA and hypersensitivity to mechanical, cold, and heat stimuli is increased in BERK sickle mice when vaso-occlusion is induced with hypoxia/reoxygenation [4, 31]. In sickle mice, but not normal mice, this induces acute sickling [45] and vaso-occlusion [46] that simulates human ischemia/reperfusion injury [47] accompanied by endothelial activation [48] and inflammation. Townes mice showed a different pattern of mechanical and thermal pain response after H/R treatment than our earlier findings with BERK sickle mice. We found that Townes mice had increased mechanical hyperalgesia and cold sensitivity after H/R treatment on day 1 that remained increased for 20 hours with further increase after H/R treatment on day 2. Heat sensitivity did not increase until 20 h after the first H/R treatment, while second H/R treatment caused an increase, which remained elevated until 7 days (Fig 5). Mechanical hyperalgesia in BERK sickle mice did not change after H/R1 but increased following H/R2, returning back to baseline after 18 h; thermal sensitivity exhibited a significant increase following both H/R treatments, returning to baseline at day 7 [4]. The pattern of deep tissue hyperalgesia in Townes mice, which increased after the H/R1 with further increase after H/R2, was similar to that previously seen with BERK-HbSS when expressed as maximum grip force recorded [4].

In our mouse colony BERK sickle mice demonstrate increased hyperalgesia as compared to age/gender-matched Townes sickle mice. However, Townes sickle mice show increased hyperalgesia as compared to their age/gender/genetics-matched controls expressing normal human hemoglobin. Importantly, Townes sickle mice provide an additional model to validate the outcomes of pain-related studies in BERK sickle mice. Our data demonstrate that relatively younger BERK sickle mice of both genders show features of pain observed in SCA, whereas relatively older Townes sickle mice show hyperalgesia. Depending upon the requirement and goals of each study, BERK or Townes mice can be used. For example, Townes sickle mice have been successfully used to express HbF [49]. Therefore studies examining both HbF induction and hyperalgesia may benefit from the use of Townes mice. These fundamental observations on pain characteristics are expected to facilitate future efforts to examine the mechanisms, identify targets, and develop pharmaceuticals to treat gender- and age-specific pain in SCA. Thus our observations will increase the translational utility of these mouse models.
